# Effects of routine repetitive transcranial magnetic stimulation on the sleep duration of patients with treatment‐resistant depression: A prospective cohort study

**DOI:** 10.1002/pcn5.187

**Published:** 2024-04-01

**Authors:** Khosro Sadeghniiat, Jayran Zebardast, Mohammadamin Parsaei, Homa Seyedmirzaei, Mohammad Arbabi, Ahmad Ali Noorbala, Sahar Ansari

**Affiliations:** ^1^ Psychosomatic Medicine Research Center Tehran University of Medical Science Tehran Iran; ^2^ Departments of Cognitive Linguistics Institute for Cognitive Science Studies (ICSS) Tehran Iran; ^3^ Maternal, Fetal, and Neonatal Research Center, Family Health Research Institute, Vali‐E‐Asr Hospital Tehran University of Medical Sciences Tehran Iran; ^4^ Sports Medicine Research Center, Neuroscience Institute Tehran University of Medical Sciences Tehran Iran

**Keywords:** insomnia, major depressive disorders, sleep, transcranial magnetic stimulation, treatment‐resistant depression

## Abstract

**Aim:**

The aim of this study was to evaluate the short‐term and long‐term effects of routine repetitive transcranial magnetic stimulation (rTMS) on the sleep duration, depressive symptoms, and quality of life of patients with treatment‐resistant depression (TRD).

**Methods:**

In this prospective cohort study, 25 participants with TRD were assessed using the Insomnia Severity Index (ISI) and four sleep duration components of the Pittsburgh Sleep Quality Index (PSQI). Depression severity was measured with Hamilton's Depression Rating Scale (HDRS) and Beck's Depression Inventory (BDI‐II), and patient‐perceived quality of life with the 36‐Item Short‐Form Survey (SF‐36). All of these measures were evaluated at baseline (T0), and immediately (T1), 6 weeks (T2), and 12 weeks (T3) after the end of intervention.

**Results:**

At T1 endpoint, HDRS, BDI, SF‐36, ISI, and three PSQI items (time to wake up, time taken to fall asleep, and Real Sleep Time) significantly improved, though these gains were reduced at follow‐up endpoints (T2 and T3). Adjusting for confounders (age, sex, occupational status, BMI, and hypnotic medication) revealed that only improvements in HDRS, BDI, and time taken to fall asleep at T1 remained statistically significant. Linear regression analyses showed no significant association between reduced time taken to fall asleep and depression symptoms, suggesting rTMS can independently enhance this parameter, irrespective of depression resolution.

**Conclusion:**

Routine rTMS therapy can potentially enhance sleep duration in TRD individuals, alongside improved depressive symptoms and quality of life. However, these benefits tend to decrease over long‐term follow‐up, emphasizing a more pronounced short‐term efficacy of rTMS.

## Graphical abstract

In this study, 25 patients with treatment‐resistant depression (TRD) received 25 daily sessions of repetitive transcranial magnetic stimulation (rTMS), alongside their ongoing medical treatment. Assessment of sleep duration (Insomnia Severity Index and Pittsburgh Sleep Quality Index), depression severity (Hamilton's Depression Rating Scale and Beck's Depression Inventory), and quality of life (36‐Item Short‐Form Survey) revealed a significant improvement in depressive symptoms and quality of life, as well as sleep duration in TRD patients immediately after the intervention. However, a diminishing trend was observed for all variables over time, suggesting a short‐term beneficial effect of rTMS on both sleep status and depressive symptoms.

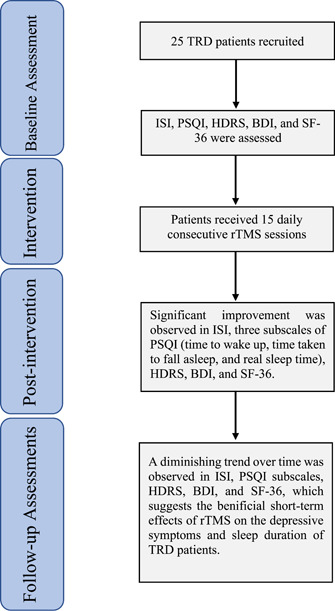

## INTRODUCTION

Repetitive transcranial magnetic stimulation (rTMS) is a noninvasive intervention that stimulates specific brain tissues by high‐ or low‐intensity magnetic fields in psychiatric disorders.^1^ These disorders include depression, bipolar disorders, psychotic disorders, anxiety disorders, and obsessive–compulsive disorder (OCD).^2^ Regarding depression, the first‐line therapy for depression consists of pharmacotherapy, especially with selective serotonin reuptake inhibitors (SSRIs), and psychotherapy.^3^ However, treatment‐resistant depression (TRD) requires more interventions, such as rTMS. In 2008, the United States Food and Drug Administration (FDA) approved rTMS as a standard therapy for TRD.^4^ rTMS mainly targets the dorsolateral prefrontal cortex, a region with reduced activity based on functional brain imaging findings.^5^ Its waves lead to an rTMS‐induced release of endogenous dopamine and serotonin neurotransmitters in prefrontal cortical areas and have been reported to improve depressive symptoms in affected people.^6^

However, few studies have investigated the effects of rTMS on insomnia in patients with TRD. Data from the Sequenced Treatment Alternatives to Relieve Depression (STAR*D) trial in the United States have reported that 84.7% of depressed people also suffer from insomnia^7^ and that insomnia can act as both a risk factor and a consequence of depression.^8^ Sleep restores and rejuvenates the brain to help maintain its optimal function.^9^ Insomnia disturbs glymphatic system functions and, therefore, the neurotoxic waste products made during wakefulness aggregate.^10^ The subsequent effects will be apparent in changes in cognition and judgment^11^ and an increased risk of neurodegenerative disorders.^12^

The effects of rTMS on depressed subjects with insomnia are discrete. In a recent study conducted by Collins et al., people with depression underwent a 6‐week course of 10‐Hz rTMS over their left dorsolateral prefrontal cortex.^13^ The results showed that depressive symptoms and sleep quality significantly improved after the intervention,^13^ similar to the findings of Nishida et al.^14^ and Lowe et al.^15^ Meanwhile, the study by Rosenquist et al. showed that rTMS did not have a significant effect on the sleep quality of individuals with TRD, although it relieved their depressive symptoms.^16^ As a result of this inconsistency, we aimed to conduct a cohort study to assess the effects of rTMS courses on people with TRD and insomnia. Our goal was to evaluate the effects of routine rTMS therapy on the sleep quantity of patients with TRD, as well as their depression severity and quality of life.

## MATERIALS AND METHODS

### Design and setting

We conducted a prospective cohort study at Imam Khomeini Hospital Complex in Tehran, Iran, from January 5, 2023, to July 10, 2023. This study encompassed all consecutive patients diagnosed with TRD and insomnia, who were referred to the psychiatry clinic between December 2, 2022, and January 2, 2023, and scheduled to undergo routine rTMS treatment. Clinical evaluation of patients was performed 1 week before the intervention (T0), immediately (2–5 days) after the intervention (T1), 6 weeks (T2), and 12 weeks (T3) after the intervention.

Ethical approval for this research was granted by the Ethics Committee and Institutional Review Borad of Tehran University of Medical Sciences (Reference Number: IR.TUMS.FNM.REC.1399.203), and we diligently adhered to the ethical principles outlined in the Declaration of Helsinki.^17^ In addition, informed verbal and written consent was obtained from all participating patients.

### Sample size calculation

The required sample size for this study was determined using the following formula. Based on findings from the study conducted by Franzen et al.,^18^ it was established that 47% of depressed patients (*p*_1_ = 0.47) experience insomnia, a percentage that decreases to 10% (*p*_2_ = 0.1) following treatment with rTMS. With a specified significance level of 5% (*α* = 0.05) for the risk of Type‐I error and a Type‐II error rate of 20% (*β* = 0.2), accounting for an additional 2% for potential loss, the optimal sample size was determined to be 25.

### Eligibility criteria

This study included individuals who met the following criteria: a confirmed diagnosis of major depressive disorder (MDD) and insomnia, as determined by an experienced attending psychiatrist following the *Diagnostic and Statistical Manual of Mental Disorders*, Fifth Edition (DSM‐V) criteria^19^; completion of a course of treatment with at least one antidepressant without significant symptom remission; age between 20 and 65 years old; the ability to participate in therapy sessions; high school diploma or higher education; proficiency in the Persian language; residency in Tehran; and no concurrent psychiatric diagnoses, as assessed by the psychiatrist's evaluation.

Patients were excluded if they expressed unwillingness to partake in the program, had been diagnosed with malignancies or autoimmune disorders, experienced significant mental distress during the study period, required hospitalization during the research period, had previously engaged in similar treatment programs, participated in another psychiatric program during the study period, modified their antidepressant medication doses or types during the research, or failed to complete the follow‐up assessments.

Throughout the duration of the study course, participants were instructed to refrain from altering their prescribed antidepressant or hypnotic medications. Additionally, they were advised against initiating any novel therapeutic interventions that could impact either depression or insomnia, including cognitive therapy.

### Procedure

All participants received the routine rTMS treatment under the direct supervision of the responsible psychiatrist. This program consisted of 15 consecutive daily sessions, excluding holidays, of 10‐Hz rTMS stimulation targeting the dorsolateral region of the prefrontal cortex within the left hemisphere. The specific stimulation sites were Brodmann areas 9 and 46, as determined by the attending physician. Each session lasted for 18.5 min and consisted of 75 trains of 40 pulsations with 120% resting motor threshold activity for a total of 3000 pulses (train time: 4 s, interval time: 11 s).

Patients were comfortably seated during each session, and earphones were employed to address the noise produced by the rTMS machine and to minimize interactions with the therapist. A researcher who had no involvement in the patients' treatment plans was responsible for collecting data from the participants at both the baseline and each subsequent follow‐up visit.

### Variables

#### Demographic and clinical variables

Data regarding age, gender, marital status, education level, employment status, recent participation in sports programs, body mass index (BMI), duration of insomnia, history of diabetes, history of hypertension, use of antidepressant medication, and utilization of medical treatments for insomnia (hypnotics) among the patients were collected at T0.

#### Depression assessment

(a) Hamilton's Depression Rating Scale (HDRS): Clinical interviews were conducted by the researcher using the 17‐item version of HDRS at T0, T1, T2, and T3.^20^ Responses to each item were assessed using a Likert scale ranging from 0 to 4. Patient scores on this scale could range from 0 to 52, with higher scores indicating a more pronounced level of depressed mood.^20^ Interpretively, total scores falling between 0 and 7 were considered within the normal range, scores of 8 to 16 signified mild depression, those between 17 and 23 suggested moderate depression, and scores exceeding 24 were indicative of severe depression.^20^
(b) Beck's Depression Inventory (BDI‐II): To assess the level of depression in patients, we utilized the BDI‐II questionnaire at T0, T1, T2, and T3.^21^ The BDI‐II comprises 21 items rated on a Likert scale ranging from 0 to 3, assessing the presence of depression over the preceding 2 weeks.^21^ Total scores on the BDI‐II range from a minimum of 0 to a maximum of 63, with higher scores reflecting a greater severity of depressive symptoms.^21^ For individuals diagnosed with depression, scores falling within the range of 0–13 indicate minimal depression, 14–19 suggest mild depression, 20–28 indicate moderate depression, and 29–63 are indicative of severe depression.^21^ In our study, the Persian‐translated version of the BDI‐II was employed, which had previously demonstrated satisfactory reliability and validity in measuring depression among individuals.^22^

#### Sleep assessment

(a) Insomnia Severity Index (ISI): To assess insomnia severity, we used the ISI questionnaire at T0, T1, T2, and T3.^23^ This tool evaluates the presence and severity of insomnia over the last month through seven items, with scores ranging from 0 to 28.^23^ Higher scores are associated with more severe insomnia.^23^ Interpretively, scores of 0–7 signify no insomnia, 8–14 indicate mild insomnia, 15–21 suggest moderate insomnia, and 22–28 denote severe insomnia.^23^ We employed the Persian‐translated ISI questionnaire, which demonstrated validity and reliability in identifying insomnia.^24^
(b) Pittsburgh Sleep Quality Index (PSQI): We utilized the initial four items of the PSQI questionnaire to evaluate the sleep duration of patients at T0, T1, T2, and T3.^25^ These items assess time to go to bed (Item 1), time to wake up in the morning (Item 2), time taken to fall sleep after going to bed (Item 3), and real sleep time (Item 4) in the past month.^25^

#### Quality of life assessment

The 36‐Item Short Form Survey (SF‐36) questionnaire was employed to assess the patient‐reported quality of life at T0, T1, T2, and T3.^26^ This questionnaire comprises 36 items that evaluate the quality of life across eight subscales: Physical Functioning, Role Physical, Bodily Pain, General Health, Vitality, Social Functioning, Role Emotional, and Mental Health.^26^ Each item is scored on a scale from 0 to 100, and the overall score is computed by taking the mean score of all 36 items, with higher scores indicating better quality of life among patients.^26^ We utilized the Persian‐translated version of the SF‐36, which has demonstrated acceptable validity and reliability in assessing the quality of life in patients.^27^

### Statistical analysis

Patients underwent assessments at baseline (T0) and three follow‐up points (T1, T2, and T3). After collecting clinical survey data at each endpoint, patient data were processed as follows:

(a) Ordinal scoring: Scores on the HDRS (normal, mild, moderate, and severe depression), BDI‐II (minimal, mild, moderate, and severe depression), and ISI (no insomnia, mild, moderate, and severe insomnia) scales were used to classify patients ordinally based on predefined categories.
(b) Continuous scoring: For the four PSQI items and SF‐36, patient scores were treated as continuous variables.

To assess the impact of the intervention on the distribution of the nonparametric ordinal variables, Wilcoxon signed‐rank test was employed. A one‐way analysis of variance (ANOVA) was utilized to compare the mean values of continuous variables across various time points during the study. Levene's test was employed to evaluate the homogeneity of variances for the mean values of each continuous variable across study endpoints. If the *p* value for Levene's test was ≥0.05, post‐hoc multiple comparisons were conducted using the Tukey test. Alternatively, if the *p* value for the Levene's test was <0.05, post‐hoc multiple comparisons were executed using the Games–Howell test.

Moreover, we performed additional analyses to adjust the effects of potential demographic cofounders (age, sex, occupational status, BMI, and hypnotic medication) on the results of our analyses. Subsequently, in instances where improvements in any sleep metrics sustained statistical significance postadjustment for all aforementioned confounders, an additional linear regression analysis was executed to evaluate whether a significant association exists between the sleep improvement and the reduction in depression symptoms measured by HDRS, or if the sleep improvement occurred independently of the resolution of depression. The statistical significance level for all analyses was set at *p* value < 0.05.

## RESULTS

### Demographics

The study cohort had a mean age of 41.2 ± 5.8 years, consisting of 15 males and 10 females. Among them, 15 were single, and 10 were married. Furthermore, seven were employed, while 18 were unemployed. The mean time since the onset of their depressive symptoms was 4.3 ± 1.8 months. The mean duration of insomnia in patients was 4.3 ± 1.8 years. Additionally, all 25 patients were undergoing treatment with antidepressant medication, with 20 out of the 25 patients reporting regular use of hypnotic medications. Further demographic details of the patients can be found in Table 1.

**Table 1:** Demographic and clinical data of the study participants.^a^

Variable	Groups	Values
Age, years		41.2 ± 5.8
Sex	Male	15 (60)
	Female	10 (40)
Marital status	Single	15 (60)
	Married	10 (40)
Education level	Diploma	13 (52)
	Bachelor	7 (28)
	Master	4 (16)
	PhD	1 (4)
Employment status	Employed	7 (28)
	Unemployed	18 (72)
Diabetes	Positive	0 (0)
	Negative	25 (100)
Hypertension	Positive	0 (0)
	Negative	25 (100)
Time passed from the onset of depressive symptoms, months		11.6 ± 4.3
Antidepressant medication	Sertraline (*n* = 10)	135.0 ± 42.4 mg/day
	Citalopram (*n* = 5)	40.0 ± 12.6 mg/day
	Escitalopram (*n* = 3)	13.3 ± 4.7 mg/day
	Bupropion (*n* = 2)	300.0 mg/day
	Venlafaxine (*n* = 2)	337.5 mg/day
	Amitriptyline (*n* = 2)	150.0 mg/day
	Mirtazapine (*n* = 1)	30.0 mg/day
Insomnia duration, years		4.3 ± 1.8
Hypnotic agent usage	Positive	20 (80)
	Negative	5 (20)
Hypnotic medication	Lorazepam (*n* = 8)	2.3 ± 0.8 mg/day
	Ramelteon (*n* = 4)	6.5 ± 1.6 mg/day
	Clonazepam (*n* = 2)	1.2 mg/day
	Diazepam (*n* = 2)	5.0 mg/day
	Doxepin (*n* = 2)	4.5 mg/day
	Quetiapine (*n* = 2)	37.5 mg/day
Recent sports program participation	Positive	0 (0)
	Negative	25 (100)
BMI		24.6 ± 1.0
Days passed from T0 to T1		11.4 ± 3.9
Days passed from T1 to T2		38.6 ± 4.8
Days passed from T2 to T3		40.2 ± 5.1

^a^ Data are presented as mean ± standard deviation or as number (percentage).

### HDRS

**Figure 1:**
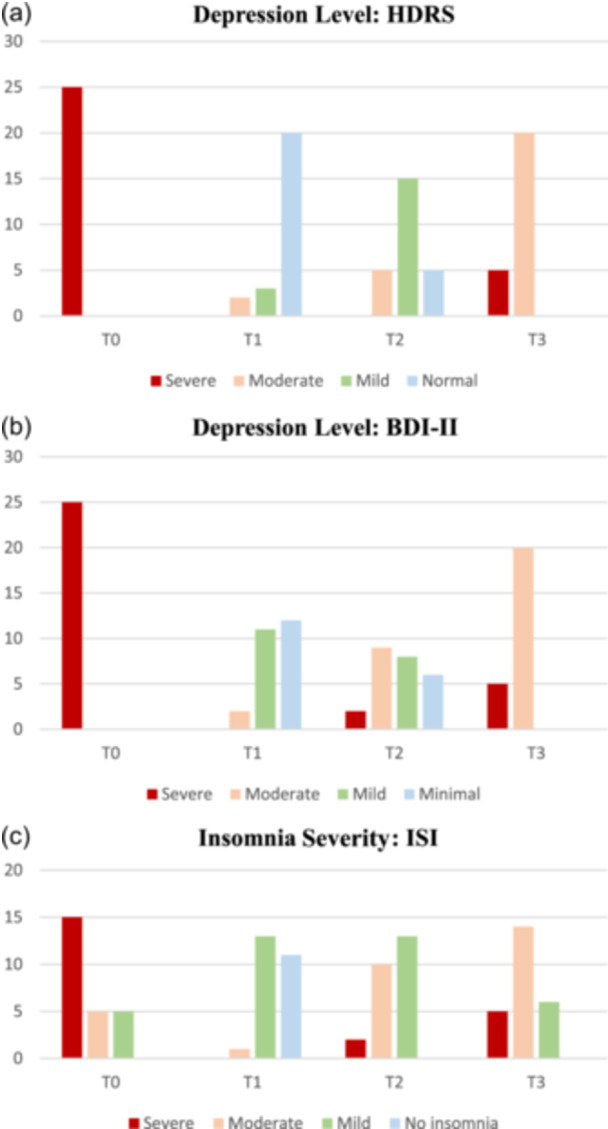
Distribution of patients with different levels of (a,b) depression and (c) insomnia across the study endpoints. BDI‐II, Beck's Depression Inventory; ISI, Insomnia Severity Index; HDRS, Hamilton's Depression Rating Scale.

At the baseline (T0), all 25 patients were classified as severely depressed according to HDRS. Immediately after the end of the intervention (T1), two patients were classified as moderately depressed, three had mild depression, and 20 were normal. At the 6‐week follow‐up (T2), five had moderate depression, 15 had mild depression, and the remaining five patients were classified as not having depression (normal). Finally, at the 12‐week follow‐up endpoint (T3), five patients were still categorized as severely depressed, while the remaining 20 patients had moderate depression (Figure 1, Table 2).

**Table 2:** Summary of the study findings.^1^

	T0	T1	T2	T3	ΔT0–T1	ΔT0–T2	ΔT0–T3	ΔT1–T2	ΔT1–T3	ΔT2–T3
*Depression level; HDRS* 2										
Severe	*n* = 25	*n* = 0	*n* = 0	*n* = 5	↑; *p* = 3.0 × 10^−6^,^ a ^,^ b ^,^ c ^,^ d ^,^ e	↑; *p* = 7.0 × 10^−6^,^ a ^,^ b ^,^ c ^,^ d ^,^ e	↑; *p* = 8.0 × 10^‐6^,^ a ^,^ b ^,^ c ^,^ d ^,^ e	↓; *p* = 2.2 × 10^−5^,^ a ^,^ b ^,^ c ^,^ d	↓; *p* = 1.0 × 10^−6^,^ a ^,^ b ^,^ c ^,^ d ^,^ e	↓; *p* = 3.0 × 10^−6^,^ a ^,^ b ^,^ c ^,^ d ^,^ e
Moderate	*n* = 0	*n* = 2	*n* = 5	*n* = 20						
Mild	*n* = 0	*n* = 3	*n* = 15	*n* = 0						
Normal	*n* = 0	*n* = 20	*n* = 5	*n* = 0						
*Depression level; BDI‐II* 2										
Severe	*n* = 25	*n* = 0	*n* = 2	*n* = 5	↑; *p* = 8.0×10^−6^,^ a ^,^ b ^,^ c ^,^ d ^,^ e	↑; *p* = 2.1×10^−5^,^ a ^,^ b ^,^ c ^,^ d ^,^ e	↑; *p* = 8.0×10^−6^,^ a ^,^ b ^,^ c ^,^ d ^,^ e	↓; *p* = 3.7×10^−5^,^ a ^,^ b ^,^ c ^,^ d	↓; *p* = 6.0×10^−6^,^ a ^,^ b ^,^ c ^,^ d ^,^ e	↓; *p* = 1.7×10^−4^,^ a ^,^ b ^,^ c ^,^ d ^,^ e
Moderate	*n* = 0	*n* = 2	*n* = 9	*n* = 20						
Mild	*n* = 0	*n* = 11	*n* = 8	*n* = 0						
Minimal	*n* = 0	*n* = 12	*n* = 6	*n* = 0						
*Insomnia level; ISI* 2										
Severe	*n* = 15	*n* = 0	*n* = 2	*n* = 5	↑; p = 6.6×10^−5^,^ b ^,^ d ^,^ e	↑; p = 4.6×10^−4^,^ b ^,^ d ^,^ e	↑; p = 0.040^,^ c ^,^ e	↓; p = 3.0×10^−4^,^ a ^,^ b	↓; p = 2.2×10^−5^,^ a ^,^ b ^,^ c	↓; p = 0.002^,^ c ^,^ d
Moderate	*n* = 5	*n* = 1	*n* = 10	*n* = 14						
Mild	*n* = 5	*n* = 13	*n* = 13	*n* = 6						
No insomnia	*n* = 0	*n* = 11	*n* = 0	*n* = 0						
*Sleep assessment; PSQI* 3										
Time to go to bed, hour	11.5 ± 0.8	11.1 ± 0.9	11.4 ± 0.8	11.5 ± 0.7	↔; *p* = 0.505	↔; *p* = 0.998	↔; *p* = 1.000	↔; *p* = 0.602	↔; *p* = 0.483	↔; *p* = 0.998
Time to wake up, hour	10.1 ± 0.9	8.8 ± 1.5	8.5 ± 1.9	9.2 ± 1.7	↑; *p* = 0.004^,^ a ^,^ b ^,^ d	↑; *p* = 0.003^,^ a ^,^ b	↔; *p* = 0.093	↔; *p* = 0.942	↔; *p* = 0.826	↔; *p* = 0.563
Time taken to fall asleep, hours	5.1 ± 1.4	1.9 ± 1.4	3.3 ± 1.4	4.2 ± 1.6	↑; *p* = 2.8 × 10^−11^,^ a ^,^ b ^,^ c ^,^ d ^,^ e	↑; *p* = 1.0 × 10^−4^,^ a ^,^ b ^,^ c	↔; *p* = 0.103^,^ b ^,^ c	↓; *p* = 0.006^,^ a ^,^ b ^,^ c ^,^ d ^,^ e	↓; *p* = 1.0 × 10^−6^,^ a ^,^ b ^,^ c ^,^ d	↔; *p* = 0.127
Real sleep time, hours	5.5 ± 1.8	7.7 ± 1.6	5.7 ± 2.0	5.5 ± 1.9	↑; *p* = 2.4 × 10^−4^,^ b ^,^ d	↔; *p* = 0.952	↔; *p* = 1.000	↓; *p* = 0.002^,^ b ^,^ c ^,^ d ^,^ e	↓; *p* = 2.4 × 10^−4^,^ a ^,^ b ^,^ c ^,^ d	↔; *p* = 0.952
*Quality of life; SF‐36* 3	55.2 ± 1.6	85.3 ± 1.1	84.2 ± 1.2	63.2 ± 0.7	↑; *p* = 6.2 × 10^−13^,^ a ^,^ b ^,^ c ^,^ d	↑; *p* = 5.9 × 10^−13^,^ a ^,^ b ^,^ c ^,^ d	↑; *p* = 6.8 × 10^−13^,^ a ^,^ b ^,^ c ^,^ d	↓; *p* = 0.007^,^ a ^,^ b ^,^ c ^,^ d	↓; *p* = 6.3 × 10^−13^,^ a ^,^ b ^,^ c ^,^ d	↓; *p* = 6.3 × 10^−13^,^ a ^,^ b ^,^ c ^,^ d

Abbreviations: BDI‐II, Beck's Depression Inventory; HDRS, Hamilton's Depression Rating Scale; ISI, Insomnia Severity Index; PSQI, Pittsburgh Sleep Quality Index; SF‐36, 36‐item Short‐Form Health Survey; ↑, significantly improved; ↔, not changed; ↓, significantly worsened.

^1^ Continuous variables are shown as mean ± standard deviation.

^2^ Nonparametric Wilcoxon signed ranks test.

^3^ Analysis of variance.

^a^ Remained statistically significant after adjusting for sex.

^b^ Remained statistically significant after adjusting for age (≤40 years and >40 years).

^c^ Remained statistically significant after adjusting for occupational status.

^d^ Remained statistically significant after adjusting for BMI (≤25 and >25).

^e^ Remained statistically significant after adjusting for hypnotic medication.

Our Wilcoxon signed‐rank analysis revealed significant improvements in the depression level of the patients immediately after the intervention compared with the baseline (*p* value < 0.001). This improvement was reduced at the 6‐ and 12‐week follow‐up endpoints (*p* value < 0.001 for ΔT1–T2, ΔT1–T3, and ΔT2–T3), however, the depression levels of the patients at 6‐ and 12‐week follow‐up endpoints were still better in comparison to the baseline (*p* value < 0.001, respectively) (Table 2).

### BDI‐II

At the baseline, all 25 patients were initially classified as severely depressed based on BDI‐II. By the first follow‐up, two patients were categorized as moderately depressed, 11 had mild depression, and 12 had minimal depression. Moving to the second follow‐up, two patients were classified as severely depressed, while nine exhibited moderate depression, eight had mild depression, and the remaining six patients displayed minimal depression. Finally, at the third follow‐up, five patients had severe depression, while the remaining 20 showed signs of moderate depression (Figure 1, Table 2).

Consistent with the HDRS scores, the depression levels within the cohort exhibited a significant improvement immediately after the intervention (*p* value < 0.001). Similar to HDRS scores, the severity of depressive symptoms consistently increased at the 6‐ and 12‐week follow‐up endpoints (*p* value < 0.001 for ΔT1–T2, ΔT1–T3, and ΔT2–T3); however, the depression level of patients at the 6‐ and 12‐week follow‐up endpoints was still lower than the baseline (*p* value < 0.001) (Table 2).

### ISI

At the baseline, 15 patients were categorized as having severe insomnia, while five had moderate insomnia according to ISI. Immediately after the intervention, none of the patients were classified as having severe insomnia. Instead, one had moderate insomnia, 13 had mild insomnia, and 11 no longer experienced insomnia. Upon reaching the 6‐week follow‐up, two patients reported severe insomnia, 10 had moderate insomnia, and 13 patients had mild insomnia. Finally, 12 weeks following the end of the intervention, five patients remained in the severe insomnia category, whereas 14 exhibited moderate insomnia, and six had mild insomnia (Figure 1, Table 2).

Through the Wilcoxon signed‐rank analysis, it was evident that the insomnia levels within the cohort displayed a significant improvement immediately after the end of the intervention (*p* value < 0.001). However, the positive effects of rTMS on the severity of insomnia in patients showed consistent reduction throughout the 6‐ and 12‐week endpoints (*p* value < 0.001 for ΔT1–T2 and ΔT1–T3 and *p* value = 0.002 for ΔT2–T3). Nonetheless, patients reported a greater amount of sleep at the 6‐ and 12‐week endpoints in comparison to the baseline (*p* value < 0.001 for ΔT0–T2 and *p* value = 0.040 for ΔT0–T3) (Table 2).

However, after adjusting the analysis for sex and occupational status of the patients, observed improvement in the ISI disappeared (Table 2).

### PSQI

**Figure 2:**
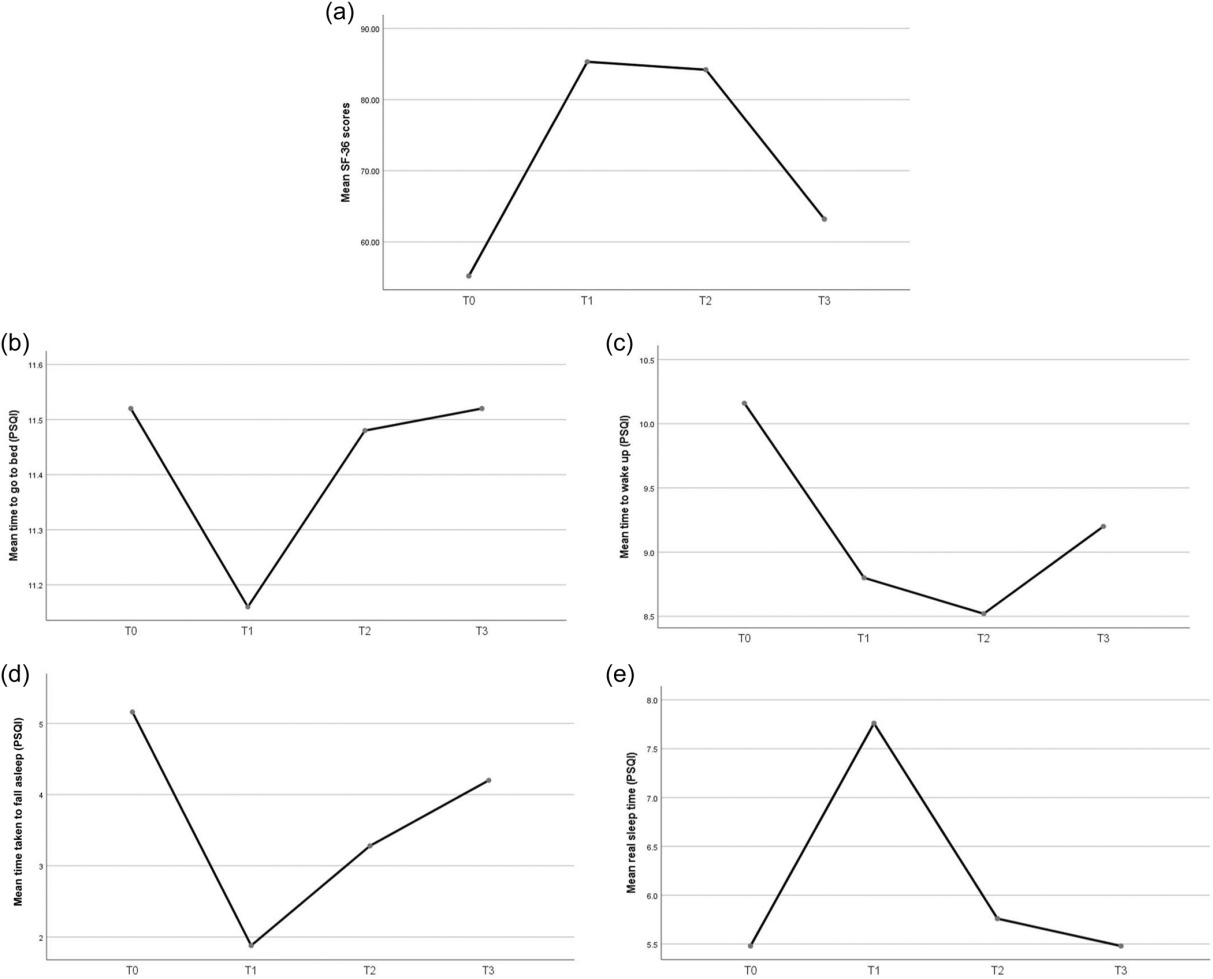
Alterations in the mean values for (a) 36‐Item Short‐Form Survey (SF‐36), and Pittsburgh Sleep Quality Index (PSQI) (b) time to go to bed, (c) time to wake up, (d) time taken to fall asleep, and (e) real sleep time throughout the study course.

At the baseline, the mean time to go to bed was 11:30 p.m. ± 48 m, and this timing remained relatively stable without any significant changes at the follow‐up endpoints throughout the study, as determined by ANOVA. On the other hand, the mean wake‐up time in the morning at the baseline was 10:06 a.m. ± 54 m. This showed significant improvements (reductions) at the T1 and T2 compared with the baseline (*p* value = 0.004 and 0.003, respectively) (Figure 2, Table 2). These improvements remained statistically significant even after adjusting the analysis for the sex and age of the patients. However, the effects of rTMS therapy disappeared after adjusting the analysis for the occupational status and the usage of hypnotic medication.

The mean time taken to fall asleep after going to bed was 5 h, 6 m ± 84 m at the baseline. The ANOVA demonstrated a significant improvement (reduction) in this variable immediately after the intervention (*p* value = 0.000). However, the positive effect of rTMS on this variable was reduced at the follow‐up points. Nevertheless, the mean time taken to fall asleep at T2 was still less when comparing with the baseline (*p* value < 0.001) (Figure 2, Table 2). The observed improvements in ΔT0–T1 maintained statistical significance even after adjusting for covariates such as sex, age, occupational status, BMI, and hypnotic medication. Subsequently, a linear regression analysis was conducted to explore potential associations between improvements in time taken to fall asleep and the resolution of depressive symptoms measured by HDRS. The results indicated no significant association between improvements in time taken to fall asleep and HDRS (*p* value = 0.855). This suggests that the improvement in this sleep parameter occurred independently of depression resolution.

Finally, the mean real sleep time of the patients was 5 h, 30 m ± 108 m at the baseline. Significant improvements (increase) in real sleep time were observed only immediately after the intervention (*p* value < 0.001) (Figure 2, Table 2). Also, the positive effect of rTMS on the real sleep time of the patients remained significant after adjusting for the age and BMI of the patients but disappeared after adjusting for the sex, occupational status, and hypnotic medication.

### SF‐36

The mean SF‐36 score of the cohort was 55.2 ± 1.6 at the baseline, which demonstrated a significant improvement after the end of the intervention (*p* value < 0.001) (Figure 2, Table 2). The patterns of alterations in the SF‐36 scores of the patients were similar to those of HDRS and BDI‐II (Figure 2, Table 2). The improvements seen in the quality of life of the patients remained significant even after controlling for age, sex, occupational status, and BMI, but disappeared after adjusting for hypnotic medication consumption.

## DISCUSSION

The current study aimed to examine the impact of rTMS on both sleep duration and depressive symptoms in individuals with TRD. The findings indicated a significant reduction in the time taken to initiate sleep and a decrease in depressive symptoms immediately after 15 consecutive daily rTMS sessions. However, the positive effects waned at the 6‐ and 12‐week follow‐ups, with no substantial differences observed in sleep status compared with the baseline. This implies that the beneficial effects of rTMS on the sleep of TRD patients are short‐term and immediate, diminishing after the conclusion of the treatment course.

As explained, insomnia and depressive symptoms similarly improve after rTMS in TRD patients. However, the exact underlying mechanisms of these effects remain to be explained. There is evidence that in most patients with successful responses to antidepressants, even SSRIs, sleep symptoms improve in parallel with other depressive signs and symptoms.^18^ This effect has also been observed in depressed people undergoing short‐term psychoanalytic psychotherapy.^28^ Meanwhile, studies have shown that cognitive‐behavioral therapy targeting insomnia can improve responses to antidepressant therapies, leading to diminished depressive symptoms.^29^ Functional studies have also indicated that prefrontal cortex activity is impaired in both depression and sleep disturbances.^30^, ^31^ As a result, the bidirectional relationship between sleep and depression makes it hard to determine if there is a cause‐and‐effect response to rTMS intervention in people with TRD.

In our longitudinal study, males comprised the majority of participants; the subjects were aged 41.2 ± 5.8 years; and their BMIs indicated them being overweight with a value of 24.6 ± 1.0. All these factors might have affected the response rates to rTMS, and the results might have significantly differed with another sample. Literature has shown that antidepressive effects of rTMS are more prominent in women, probably due to closer proximity of the brain to the scalp at the prefrontal cortex (the rTMS target), more dense grey matter and gyrification in the prefrontal cortex, and high levels of estradiol, which enhances the cortical excitability.^32^ Moreover, a recent systematic review and meta‐analysis has shown that a older age is associated with greater improvements in depressive symptoms after rTMS.^33^ Regarding BMI, a study has reported that remission rates of people with obesity (BMI ≥ 30) are greater than those without obesity. Plus, obese depressed people show a shorter time to remission.^34^

Electroencephalographic assessments in depression and concurrent sleep disturbances have indicated disinhibition of rapid eye movement (REM) sleep, altered sleep continuity, and impaired non‐REM sleep in affected people.^35^ Herein, we did not assess electroencephalographic changes in the included participants. In a previous study by Pellicciari et al., depressed patients underwent 10 daily sessions of sequential bilateral rTMS.^36^ They reported induced topographical‐specific reductions in the alpha activity during REM sleep over the left dorsolateral prefrontal cortex, which was significantly correlated with the clinical outcome.^36^ In another similar study, Saeki et al. aimed to target changes in slow‐wave activity and sleep spindle activity in depressed people undergoing five sessions of rTMS.^37^ Their work highlighted a local increase of slow wave activity during non‐REM sleep and no change in REM sleep parameters in the left dorsolateral prefrontal cortex.^37^ More studies assessing polysomnographic electroencephalographic changes might better unravel mechanisms of sleep improvement in people with TRD.

As previously explained in the introduction, various studies have been conducted to find the effects of rTMS on concurrent depression and sleep disturbances so far. In a study by Collins et al., patients underwent 30 sessions of 10‐Hz rTMS in 6 weeks on the left dorsolateral prefrontal cortex.^13^ The results showed that depressive and sleep difficulties improved significantly after intervention (Patient Health Questionnaire‐9 *p* value < .0001 and PSQI *p* value = 0.01).^13^ Similarly, Grunhaus et al. did the same protocol in 20 sessions on people with TRD and achieved improved results on depression and insomnia (HRDS and PSQI *p* values < 0.05).^38^ They also reported that the responses of patients undergoing rTMS and electroconvulsive therapy do not significantly differ in this regard.^38^ In another study, Nishida et al. administered 10 sessions of bilateral rTMS on the dorsolateral prefrontal cortex (1 Hz on the right and 10 Hz on the left).^14^ They similarly reported significant improvements (PSQI *p* value = 0.004 and HRDS = 0.002).^14^ Nevertheless, in a sham‐controlled study by Rosenquist et al., depressed subjects did not show improved sleep quality by HRDS sleep‐factor and Depressive Symptoms Self‐Report sleep‐factor scores after 30 sessions of 10‐Hz left dorsolateral prefrontal cortex rTMS (*p* values 0.10 and 0.85, respectively).^16^ Sham TMS can act as a placebo to its active form and is applied by increasing current to target strength and tapering off for several seconds afterward.^39^ Different protocols regarding session numbers and the targeted regions, sex and age profiles of subjects, BMI and their body compositions, and drug history of hypnotic antidepressants might explain the observed differences in these studies.

The results of our study should be interpreted in light of some limitations. The first is the limited sample size and the second is the absence of a control group. Sham rTMS could be considered as an intervention for a control group of TRD patients. Moreover, the exact type of antidepressants and hypnotic drugs TRD patients were taking during the trial might have affected the results, about which the sample was not homogenous. Despite these constraints, our study offers preliminary evidence suggesting potential positive effects of rTMS on the sleep status of individuals with TRD. To enhance the robustness of these findings, future studies with larger, multicentered samples and the inclusion of a sham rTMS control group are recommended. This approach would better elucidate and replicate the outcomes observed in our study.

Collectively, our findings indicate that the implementation of routine rTMS therapy can potentially improve the sleep duration of individuals with TRD, in addition to improving their depressive symptoms and overall quality of life. It is noteworthy, however, that these positive effects appear to wane over the course of long‐term follow‐up assessments, suggesting a more pronounced short‐term efficacy of rTMS. To gain a comprehensive understanding of the precise impact of rTMS on the sleep patterns of TRD patients, it is imperative to undertake further studies encompassing larger sample sizes and incorporating control groups.

## AUTHOR CONTRIBUTIONS

**Khosro Sadeghniiat**: Conceptualization; supervision; writing—review and editing. **Jayran Zebardast**: Conceptualization; writing—original draft. **Mohammadamin Parsaei**: Writing—original draft; formal analysis. **Homa Seyedmirzaei**: Writing—original draft. **Mohammad Arbabi**: Writing—review and editing. **Ahmad Ali Noorbala**: Writing—review and editing. **Sahar Ansari**: Supervision; writing—review and editing.

## DISCLOSURE STATEMENT

The authors of this paper declare no financial biomedical interests or potential conflitcs of interest.

## CONFLICT OF INTEREST STATEMENT

The authors declare no conflicts of interest.

## ETHICS APPROVAL STATEMENT

Ethical approval for this research was granted by the Ethics Committee and Institutional Review Borad of Tehran University of Medical Sciences (Reference Number: IR.TUMS.FNM.REC.1399.203), and we diligently adhered to the ethical principles outlined in the Declaration of Helsinki.

## PATIENT CONSENT STATEMENT

Informed verbal and written consent were obtained from all participating patients.

## CLINICAL TRIAL REGISTRATION

Not applicable.

## Data Availability

The data that support the findings of this study are available from the corresponding author upon reasonable request.
